# The bioeroding sponge *Cliona orientalis* will not tolerate future projected ocean warming

**DOI:** 10.1038/s41598-018-26535-w

**Published:** 2018-05-29

**Authors:** Blake D. Ramsby, Mia O. Hoogenboom, Hillary A. Smith, Steve Whalan, Nicole S. Webster

**Affiliations:** 1 College of Science and Engineering and ARC Centre of Excellence for Coral Reef Studies, James Cook University, Townsville, Queensland Australia; 2 Australian Institute of Marine Science, Townsville, Queensland Australia; 3 AIMS@JCU, Australian Institute of Marine Science and James Cook University, Townsville, Queensland Australia; 4 Marine Ecology Research Centre, School of Environment, Science and Engineering, Southern Cross University, Lismore, New South Wales Australia; 5 Australian Centre for Ecogenomics, The University of Queensland, Brisbane, 4072 Queensland Australia

## Abstract

Coral reefs face many stressors associated with global climate change, including increasing sea surface temperature and ocean acidification. Excavating sponges, such as *Cliona* spp., are expected to break down reef substrata more quickly as seawater becomes more acidic. However, increased bioerosion requires that *Cliona* spp. maintain physiological performance and health under continuing ocean warming. In this study, we exposed *C. orientalis* to temperature increments increasing from 23 to 32 °C. At 32 °C, or 3 °C above the maximum monthly mean (MMM) temperature, sponges bleached and the photosynthetic capacity of *Symbiodinium* was compromised, consistent with sympatric corals. *Cliona orientalis* demonstrated little capacity to recover from thermal stress, remaining bleached with reduced *Symbiodinium* density and energy reserves after one month at reduced temperature. In comparison, *C. orientalis* was not observed to bleach during the 2017 coral bleaching event on the Great Barrier Reef, when temperatures did not reach the 32 °C threshold. While *C. orientalis* can withstand current temperature extremes (<3 °C above MMM) under laboratory and natural conditions, this species would not survive ocean temperatures projected for 2100 without acclimatisation or adaptation (≥3 °C above MMM). Hence, as ocean temperatures increase above local thermal thresholds, *C. orientalis* will have a negligible impact on reef erosion.

## Introduction

Increasing global temperatures are requiring organisms to acclimate to greater thermal extremes, migrate, or suffer reduced fitness and, potentially, local extirpation. The earth’s climate is already estimated to be 0.85 °C warmer than it was in 1880, which is affecting both terrestrial and marine ecosystems^1^. Much of the thermal energy (~60%) associated with warming has been absorbed by the oceans, resulting in melting sea ice, rising sea levels^1^, and record temperatures in tropical waters^2,3^. Ocean warming has already resulted in extensive coral mortality^3,4^, as evidenced in 2015/2016, when extreme temperatures led to consecutive mass coral bleaching events around the world^2,3^.

Corals contain photosynthetic dinoflagellates (genus *Symbiodinium*) that provide them with organic carbon^5^. However, the symbiosis is thermally sensitive and exposure to elevated temperature disrupts *Symbiodinium* photosynthesis^6^ and causes coral bleaching^7,8^. Some coral species and *Symbiodinium* ‘types’ are more thermally tolerant than others^9–11^, but even tolerant genotypes can be overwhelmed by severe temperature stress^3^. Nonetheless, in some cases, previous exposure to high temperature or association with tolerant *Symbiodinium* can lead to greater thermal tolerance of the coral symbiosis^9,10,12,13^ and accelerate recovery following bleaching^9,14,15^.

In comparison to reef-building corals, sponges are thought to be relatively tolerant of increasing sea surface temperatures^16,17^. In particular, some bioeroding sponges can tolerate temperatures that induce bleaching in sympatric corals^18–22^. Bioeroding sponges, principally the genus *Cliona*, are important members of coral reef communities as they erode the limestone substratum by reducing the pH at the sponge:substratum interface^23^, dissolving the substratum, and extracting microscopic ‘chips’ of calcium carbonate^24,25^. Like corals, many bioeroding sponge species form symbioses with photosynthetic *Symbiodinium* and photosynthesis enhances their growth and bioerosion^26–28^. However, while dependence on *Symbiodinium* may increase the thermal sensitivity of *Cliona*, little is known about how these sponges will tolerate predicted incremental temperature increases or whether they can recover from extreme thermal stress^29,30^.

Experimental research combining elevated temperature and reduced pH has shown that sponge bioerosion rates will likely increase under conditions of ocean acidification^31–35^. However, warming can have negative effects on bioeroding sponges, including bleaching or necrosis; and it is likely that these negative effects will override all other environmental factors^29,30,32^. For instance, under temperature and pH conditions predicted for 2100, the bioeroding sponge *Cliona orientalis* bleaches, and the associated reduction in photosynthetic productivity results in a negative energy budget for the sponge despite accelerated rates of erosion^29,34^. Warming was subsequently identified as the primary stressor inducing bleaching in a bioeroding sponge^30^. However, temperature tolerance appears to vary among bioeroding sponge species as bleaching or mortality was not observed in all studies^31,36^. Therefore, the net effect of climate change on bioeroding sponges, and on their erosion rates, appears to be species-specific.

Identifying thermal thresholds under near-future warming requires measurement of performance across a broad range of incremental temperature changes. This incremental approach enables a holistic understanding of temperature effects by allowing quantification of the optimal temperature for peak physiological performance, along with derivation of sub-lethal and lethal temperature thresholds^37,38^. A similar approach has been applied to corals to quantify adaptation to local thermal regimes^39^ and to determine how coral respiration and photosynthesis varies with temperature^40^. Here, we experimentally assessed the ability of *C. orientalis* to tolerate incrementally increasing sea surface temperatures between 23–32 °C, which represents the annual temperature range for the studied sponges (22–30 °C) and warmer temperatures predicted for 2100 (31, 32 °C)^1^. In addition, we monitored recovery from temperature stress to evaluate the impact of thermal exposure on sponge survival. Photosynthetic performance of *Symbiodinium* and energy reserves of the sponge were quantified to identify temperature optima and define thermal thresholds. To contextualize the laboratory experiment, we assessed bleaching severity for *C. orientalis* during the 2017 mass coral bleaching event.

## Results

### Response to laboratory thermal exposure

#### Bleaching and *Symbiodinium* identity

**Figure 1:**
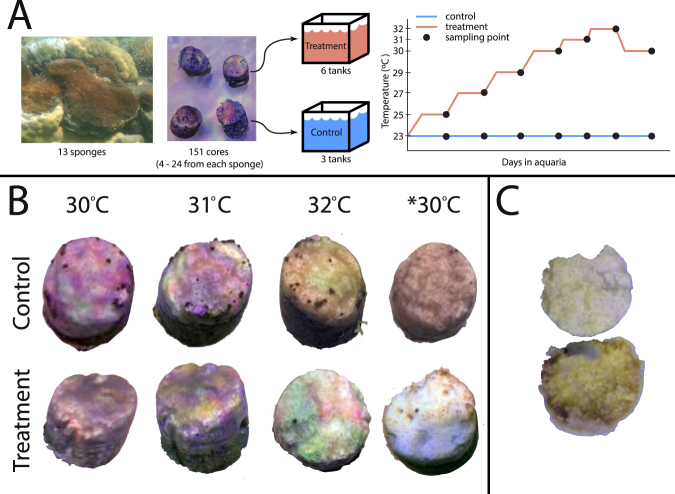
(**A**) Sponge sampling and temperature treatments. Healthy and bleached *Cliona orientalis* cores. (**B**) A time-series of two cores from the control and heated treatments, respectively. Cores in the heated treatment visibly bleached at 32 °C and had not recovered four weeks later at *30 °C. (**C**) The top surface and pinacoderm of a bleached core at the end of the experiment. The pinacoderm of bleached cores (interior) remained healthy despite the absence of *Symbiodinium* for four weeks.

*Cliona orientalis* survived temperatures up to 31 °C with no visual signs of bleaching and little evidence of compromised health, such as discolouration or tissue regression (Fig. 1B). However, at 32 °C, more than 50% of the cores became visibly bleached within three to five days and this increased to 70% bleached after 8 days (Fig. 1B). The condition of some cores also visibly deteriorated in the four weeks following bleaching, including the presence of algae at the margin of the cores (Fig. 1C). The narrow bleaching threshold of 32 °C identified for *C. orientalis* is 2.9 °C above the maximum monthly mean at Orpheus Island. A low level of mortality occurred in both temperature treatments but was constrained to cores from a few specific sponge genotypes.

Only a single *Symbiodinium* ITS2 type was associated with *C. orientalis*, regardless of temperature treatment or bleaching state. ITS2 sequences clustered into 61 OTUs at 97% sequence similarity, but only 21 of these matched the *Symbiodinium* database, and only 9 were confidently matched with bitscores greater than 100. Of the nine *Symbiodinium* OTUs, one OTU comprised 96% of the *Symbiodinium* sequences and was the most abundant OTU in every sponge core. The ITS2 sequence of the dominant OTU was identical to *Symbiodinium* clade G previously sequenced from *C. orientalis* (Genbank accession JQ247051) and which was recently described as *Symbiodinium endoclionum*^41^. One other OTU occurred in 97% of the cores and was also most similar to *Symbiodinium* clade G from *C. orientalis*, differing from the dominant ITS2 sequence by one insertion of eight nucleotide substitutions. The remaining *Symbiodinium* OTUs were most similar to *Symbiodinium* clades A, B, or C, however these OTUs comprised <1% of total sequences and occurred in <20% of the samples.

#### Photosynthesis and respiration

**Figure 2:**
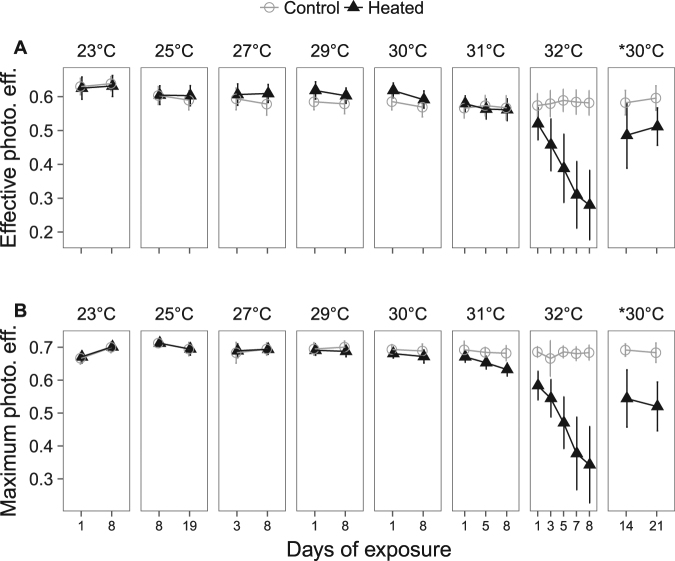
Photochemical efficiency of *Symbiodinium* within *C. orientalis*. Effective (**A**) and maximum (**B**) photochemical efficiencies are shown for control cores measured at 23 °C (grey open circles and lines) and heated cores at elevated temperature (black triangles and lines). Panels separate the temperature of the heated treatment. Points represent means and error bars indicate one SD.

**Figure 3:**
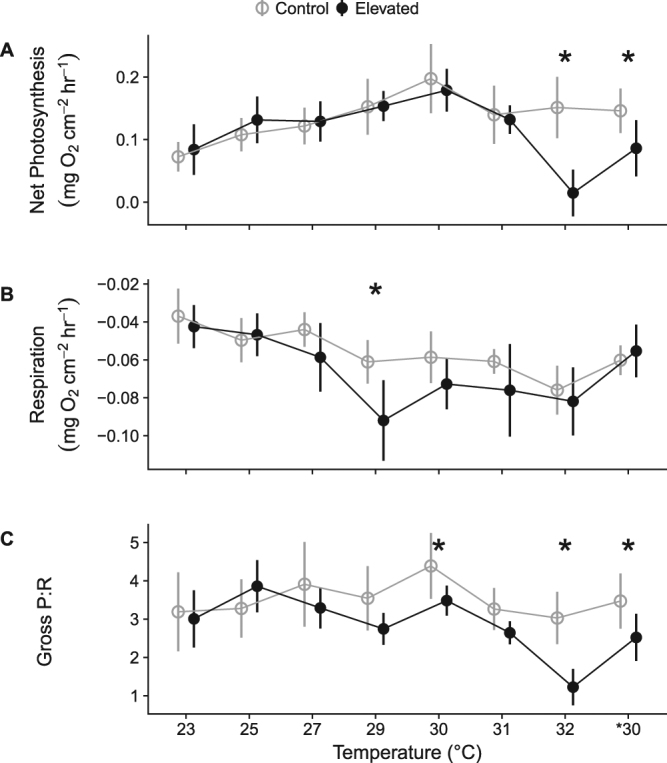
Oxygen flux rates for net photosynthesis (**A**), respiration (**B**), and the ratio of gross photosynthesis to respiration (**C**) for *C. orientalis* between 23 and 32 °C. Control cores (grey open circles and lines) were all measured at 23 °C, while heated cores (black circles and lines) were sampled at the temperature indicated in the legend. *30 indicates samples that were exposed to 32 °C and then returned to 30 °C for four weeks following bleaching. Points represent means and error bars indicate one standard error. Asterisks indicate temperature increments where sponges in control and heated treatments had significantly different responses.

Temperature effects were interpreted as significant temperature*treatment interactions which indicate the temperatures at which heated sponge cores responded differently than control cores (Table 3). Photosynthesis was largely unaffected by temperatures up to and including 31 °C, but became inhibited at 32 °C. Sponges at 27 and 29 °C had higher effective (∆F/F_m_’), but not maximum (F_v_/F_m_), photochemical efficiency than sponges maintained at 23 °C (Fig. 2; eff. 27 °C: z = −4.5, *p* < 0.01; eff. 29 °C: z = −3.2, *p* = 0.01). At 29–31 °C, sponges had significantly lower maximum photochemical efficiencies than control sponges (*p* < 0.01), but the differences were small, and *C. orientalis* maintained 93% of maximum photochemical efficiency of control sponges at 31 °C. However, at 32 °C, maximum and effective photochemical efficiencies were 52% and 50% of control sponges, respectively (Table 4). As with photochemical efficiency, the photosynthetic rate (gross oxygen production) did not differ from controls at temperatures up to and including 31 °C (Fig. 3A; *p* > 0.86) but was 43% lower than controls in sponges exposed to 32 °C (Table 4).

In contrast to photosynthesis, the effect of thermal exposure on sponge respiration was greatest at 29 °C, where heated sponges had 47% higher respiration rates than control sponges (Fig. 3B; z = −3.4, *p* < 0.01). At temperatures close to 29 °C (27, 30, and 31 °C), respiration was 28–35% higher than controls, but these differences were not significant (0.09 < *p* < 0.29). For sponges at 32 °C, respiration rates were similar to control sponges (Table 4). The ratio of gross photosynthesis to respiration (P/R) was affected at a similar temperature as the respiration rates (Fig. 3C). Sponges at 29, 30, and 31 °C had 79% of the sponge P/R of control sponges, coinciding with faster respiration rates (Fig. 3B), but the difference was only statistically significant at 30 °C (z = 3.1, *p* = 0.01). Sponges at 32 °C had 37% of the P/R of control sponges (Table 4), indicating a loss of productivity of the symbiosis.

#### Sponge condition

**Figure 4:**
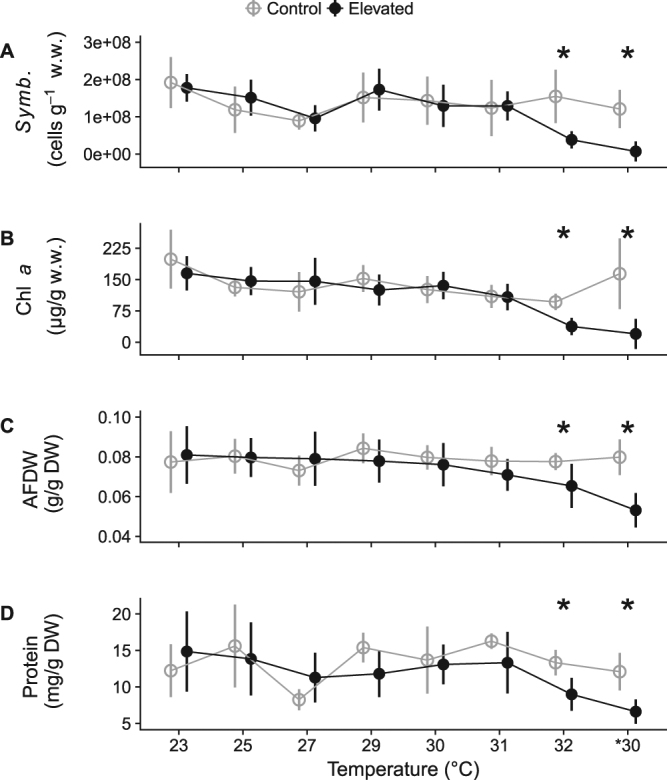
Tissue contents of *Symbiodinium* (**A**), chlorophyll *a* (**B**), ash-free dry weight (**C**; AFDW), and protein content (**D**) for *C. orientalis* between 23 and 32 °C. Controls (grey open circles and lines) were all sampled at 23 °C while heated cores (black circles and lines) were sampled at the temperature indicated in the legend. *30 indicates samples that were exposed to 32 °C, bleached, and were returned to 30 °C for four weeks. Points represent means and error bars indicate standard error. Asterisks indicate temperature increments where cores in control and heated treatments had significantly different responses.

Temperatures less than 32 °C did not significantly affect the condition of *C. orientalis* and the density of *Symbiodinium*, chlorophylls *a* and *c*_2_, protein, and organic matter were similar to control sponges (Fig. 4; *p* > 0.20). At 32 °C, the bleached sponges contained 25% of the *Symbiodinium*, 35% of chlorophyll *a*, and 42% of chlorophyll *c*_2_ of the control sponges (Table 4). Moreover, sponges at 32 °C contained 83% of the organic matter and 66% of the protein found in control sponges (Table 4), signifying reduced condition of the sponge.

While the *a*:*c*_2_ ratio decreased over the course of the experiment (Table 3, Temperature), it was not strongly affected by temperature, as heated sponges were 91–107% that of control sponges throughout the experiment and differences between treatments were not significant (Table 3, Treatment:Temperature).

### Recovery from laboratory thermal exposure

#### Bleaching and* Symbiodinium* identity

The *Symbiodinium* associated with *C. orientalis* did not change following bleaching, with all cores dominated by *Symbiodinium endoclionum*. Four weeks following bleaching, *C. orientalis* cores appeared white, and both the *Symbiodinium* density and chlorophyll content revealed that the sponges had not recovered from their bleached state, indicating prolonged holobiont disruption (Fig. 4).

#### Photosynthesis and respiration

*C. orientalis* recovered some photosynthetic capacity when returned to *30 °C, with photochemical efficiencies, photosynthetic rates, and P/R being higher than when sponges were at 32 °C (Fig. 3, Table 4). However, this response was likely due to other photosynthetic colonisers as *Symbiodinium* densities remained low in sponges at *30 °C (Fig. 3). Regardless, recovery of photosynthesis was incomplete, as photochemical efficiencies, photosynthetic rates, and P/R remained lower than control sponges (Fig. 3, Table 4). The respiration rates of sponges returned to *30 °C were higher than the sponges at 32 °C, but not significantly different from controls (Fig. 3, Table 4).

#### Sponge condition

Tissue contents indicated that the condition of the 32 °C sponges continued to deteriorate after they were returned to *30 °C (Fig. 4, Table 4). Chlorophyll content, organic matter, and protein content were lower in sponges at *30 °C than at 32 °C, but *Symbiodinium* density was not significantly different (Table 4). In addition, all measured tissue contents remained lower in sponges returned to *30 °C than control sponges (Fig. 4, Table 4).

### Field bleaching surveys

**Figure 5:**
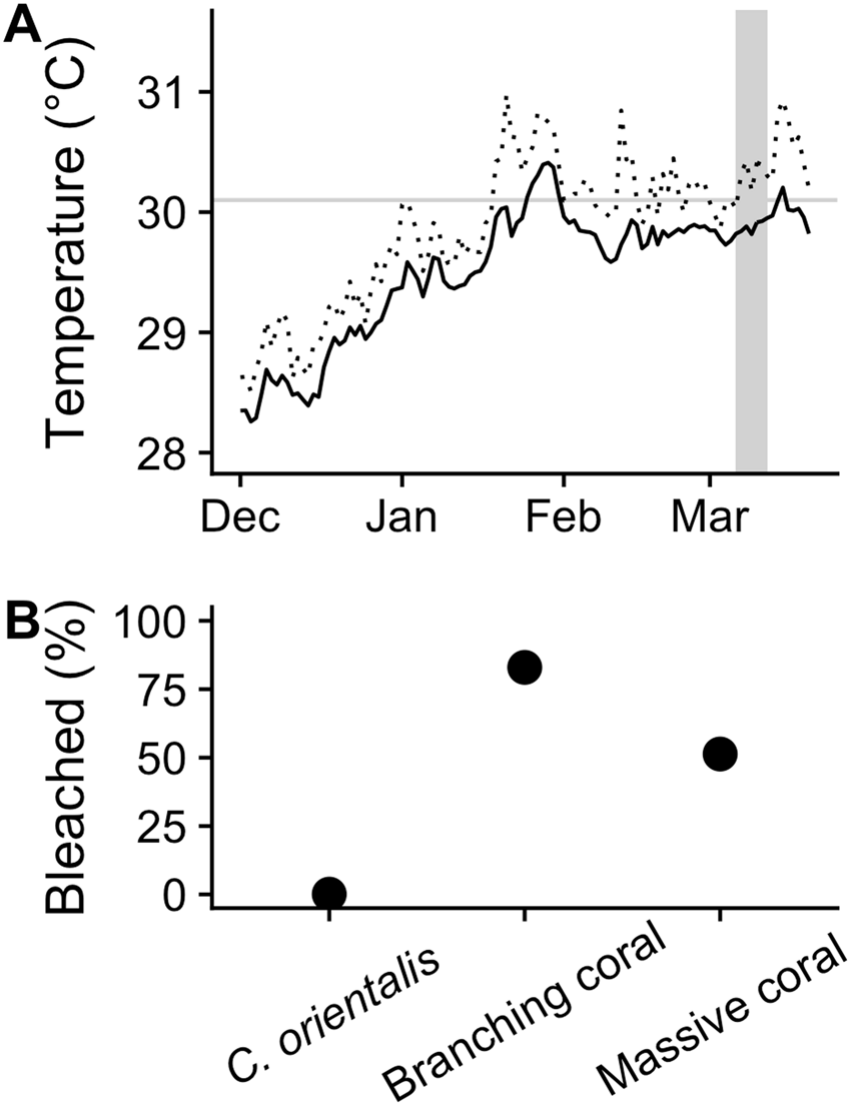
The temperature (**A**) and bleaching severity (**B**) during a natural bleaching event in the Palm Islands, Great Barrier Reef, Australia in February-March 2017. The daily mean (solid black line) and maximum (dotted line) temperature for the twelve weeks preceding the surveys are shown. The horizontal grey line indicated the local coral bleaching threshold (30.1 °C) and the vertical grey bar denotes the survey period.

A total of 133 *C. orientalis* sponges, 1891 branching corals, and 1068 massive corals were counted among the six survey sites. No bleached *C. orientalis* sponges were observed in any of the video transects. In contrast, 83% (±6.0 SD) of branched coral colonies and 51% (±3.2 SD) of massive coral colonies were bleached (Fig. 5B).

Temperatures did not reach 32 °C during the 2017 bleaching event at Orpheus Island (Fig. 5A). During the two weeks preceding the surveys, daily mean temperatures averaged 29.8 °C at 5.8 m depth (Source: Australian Institute of Marine Science; http://data.aims.gov.au) and, during the 12 weeks preceding the surveys, the cumulative thermal exposure summed to 1 degree heating week (weeks above 30.1 °C). In comparison, the laboratory experiment indicated that *C. orientalis* bleached at 32 °C after accumulating 2.5 degree heating weeks (3 days at 32 °C; Table 1).

**Table 1:** Target and actual temperatures during the laboratory experiment.

Time point	Target temperature	Heated (n = 6)	Control (n = 3)	°C above MMM + 1	Accumulated DHW
17	23.0	23.5 ± 0.1	23.4 ± 0.2	0	0
43	25.0	25.2 ± 0.1	23.1 ± 0.1^#^	0	0
56	27.0	27.0 ± 0.1	23.3 ± 0.1	0	0
71	29.0	29.0 ± 0.2	23.0 ± 0.2	0	0
84	30.0	30.0 ± 0.1	23.1 ± 0.1	0	0
96	31.0	30.9 ± 0.1^#^	23.2 ± 0.1	0.8	1.3
108	32.0	32.0 ± 0.1	23.1 ± 0.2	1.9	4.4
144	*30.0	30.1 ± 0.1	23.1 ± 0.3	0	4.4

For each target temperature, tank temperatures were recorded every 5 minutes over 8 days (mean ± SD°C). ^#^Indicates where the SD was less than 0.05 °C. °C above the maximum monthly mean (MMM) at Orpheus island (29.1 + 1.0 °C) indicates the thermal anomaly above long-term summer temperatures which was used to calculate the accumulated degree heating weeks (DHW) as the product of the thermal exposure and the thermal duration (°C-weeks).

**Table 2:** The number of cores and original sponges sampled for oxygen flux and tissue contents at each timepoint. *30 indicates the recovery period at 30.0 °C following exposure to 32 °C.

Time point	Target temperature	Heated		Control	
		Sponges	Cores	Sponges	Cores
17	23.0	9	9	6	6
43	25.0	10	12	6	6
56	27.0	9	12	6	6
71	29.0	9	12	6	6
84	30.0	8	12	6	6
96	31.0	7	12	5	6
108	32.0	8	16	4	5
144	*30.0	8	16	4	6

**Table 3:** Statistical results of the laboratory experiment. Parameters tested include maximum photochemical efficiency (F_v_/F_m_), and effective photochemical efficiency (∆F/F_m_’), photosynthetic rate (P), respiration rate (R), the ratio P:R, *Symbiodinium* density (*Symb*.), chlorophyll *a* and *c* density (chl), protein content, and ash-free dry weight (AFDW).

Trans.		Out. rem.	Treatment (num. df = 1)					Temperature (num. df = 7)					Treatment: Temperature (num. df = 7)				
			SS	MS	D. df	F	*p*	SS	MS	D. df	F	*p*	SS	MS	D. df	F	*p*
***Photosynthesis***																	
F_v_/F_m_	p/(1 − p)	N	18.9	18.9	686.1	676.4	<0.01	35.5	5.1	679.0	181.9	<0.01	25.1	3.6	677.9	128.5	<0.01
∆F/F_m_’	p/(1 − p)	N	0.7	0.7	9.8	106.2	<0.01	23.1	3.3	674.0	106.2	<0.01	11.3	1.6	672.5	52.0	<0.01
Gross P	—	N	<0.1	<0.1	121.4	4.0	<0.01	0.3	<0.1	24.1	128.3	<0.01	<0.1	<0.1	7.6	124.7	<0.01
R	—	Y	<0.1	<0.1	121.0	18.9	<0.01	<0.1	<0.1	128.8	14.9	<0.01	<0.1	<0.1	125.0	3.0	0.01
Gross P:R	—	Y	14.3	14.3	119.9	41.3	<0.01	25.3	3.6	10.5	126.2	<0.01	12.9	1.8	5.3	122.8	<0.01
***Sponge condition***																	
*Symb*.	—	N	18944	18944	125.2	8.5	<0.01	173824	24823	129.7	11.1	<0.01	89443	12778	127.4	5.7	<0.01
Chl *a*	—	N	25624	25624	123.2	19.6	<0.01	121098	17300	13.2	128.4	<0.01	94888	13556	10.4	13556	<0.01
Chl *c*	—	N	990	990	123.1	20.3	<0.01	7072	1010	20.8	128.1	<0.01	4372	624.6	12.8	125.2	<0.01
Chl *a*:*c*	Log	Y	<0.1	<0.1	123.3	0.4	0.52	0.8	0.1	5.3	128.6	<0.01	0.1	<0.1	0.8	126.1	0.59
Protein	Log	N	0.8	0.8	120.5	10.4	<0.01	4.0	0.6	7.9	130.2	<0.01	2.3	0.3	4.6	124.8	<0.01
AFDW	Log	Y	0.3	0.3	122.1	14.3	<0.01	0.7	0.1	5.8	129.2	<0.01	0.7	0.1	5.5	125.0	<0.01

Units for each measure are provided in Figs 3–4. Data were analysed using a linear mixed model with four components: temperature treatment, temperature, treatment:temperature interaction, and a random intercept for the *C. orientalis* sponge. *Symbiodinium* density was analysed with an additional random intercept for each aquarium. Parameters that were significantly affected by the temperature exposure have a significant treatment:temperature interaction term. The table includes the transformation used (Trans.), whether any outlying observations were removed, and statistical estimates: sums of squares (SS), mean square (MS), denominator degrees of freedom (D. df), F ratio (F), and P value (*p*).

**Table 4:** Results of post-hoc comparisons.

	Exposure				Recovery					
	32 °C: Heated vs Ctrl.				Heated: *30 vs 32 °C		*30 °C: Heated vs Ctrl			
	Min. sig. temp		z	*p*		z	*p*		z	*p*
***Photosynthesis***										
F_v_/F_m_	29	C > 32	27.8	<0.01	*30 > 32	11.3	<0.01	C > H	14.2	<0.01
∆F/F_m_’	32	C > 32	15.8	<0.01	*30 > 32	12.2	<0.01	C > H	5.4	<0.01
Gross P	32	C > 32	7.8	<0.01	*30 > 32	−5.6	<0.01	C > H	3.7	<0.01
R	—	—	−0.8	0.99	*30 > 32	−4.8	<0.01	—	1.2	0.89
Gross P:R	30	C > 32	6.1	<0.01	*30 > 32	5.8	<0.01	C > H	3.2	<0.01
***Sponge condition***										
*Symb*.	32	C > 32	4.3	<0.01	—	1.9	0.42	C > H	5.3	<0.01
Chl *a*	32	C > 32	3.7	<0.01	32 > *30	12.8	0.33	C > H	8.6	<0.01
Chl *c*	32	C > 32	3.9	<0.01	32 > *30	3.33	<0.01	C > H	9.5	0.01
Chl *a*:*c*	—	—	—	—	—	—	—	—	—	—
Protein	32	C > 32	3.0	0.03	32 > *30	3.2	0.01	C > H	4.8	<0.01
AFDW	32	C > 32	2.8	0.05	32 > *30	4.3	<0.01	C > H	6.6	<0.01

To compare the sensitivity of different parameters, the table includes the lowest temperature increment with a significant difference between heated and control sponges. While post-hoc tests were used to test for differences after each temperature increase, detailed results are presented from 3 post-hoc tests that indicate 1) whether cores heated to 32 °C differed from control cores, 2) whether cores heated to 32 °C differed from cores returned to *30 °C (i.e., recovery), and 3) whether cores reduced to *30 °C differed from controls (i.e., recovery). For each test, the table indicates the direction of the difference between groups, the z value, and *p* value. *p* values were corrected for multiple comparisons using a single-step correction. – indicates where there was no difference between treatments. Parameter abbreviations are listed with Table 3.

## Discussion

Bioeroding sponges are generally thought to be tolerant of environmental stressors, including elevated temperature, ocean acidification, and eutrophication, raising concerns about increased reef erosion under future projected climate scenarios^42,43^. Here, we show that incremental increases in ocean temperature up to 30 °C have negligible effects on *C. orientalis*, but *C. orientalis* bleaches when exposed to 32 °C, and exhibits little potential for recovery. At the collection site, 32 °C represents an increase of 3 °C above the maximum monthly mean temperature and corresponds to the increase expected under very high greenhouse gas emissions by 2100, but could represent the mean temperature as soon as 2078 (RCP 8.5)^1^, suggesting that *C. orientalis* could bleach regularly by the end of this century. The results of this study do not support the hypothesis that bioeroding sponges (particularly those species with photosynthetic symbionts) will play a larger role in structuring future reefs.

Temperature exposure in the laboratory revealed a narrow thermal threshold for *C. orientalis*, with sponges appearing visibly healthy after 10 days at 31 °C, but bleaching after only 3 days at 32 °C. This narrow threshold is similar to several sympatric coral species that bleached following 1 °C temperature increases between 31 and 33 °C^44^. In thermally sensitive corals, bleaching coincides with reduced condition and growth^7^ and *C. orientalis* exhibited similar negative responses, including a 75% reduction in *Symbiodinium* density, 17% reduction in organic matter and 44% reduction in protein content of *C. orientalis*. Few bleached cores exhibited necrosis which had been previously reported in *C. orientalis* from Orpheus Island after exposure to only 2 °C above MMM. The discrepancy between studies likely results from the faster temperature increases or acute exposures (3–72 h) used in previous research^32,45^. The thermal threshold identified here is consistent with findings for *C. orientalis* in the southern GBR, which tolerates exposure to +2.0 °C above MMM (27.3 °C)MMM = 27.3 °C^34^, but bleaches at +2.7 °C^30^, and dies at +3.5 °C^34^. Taken together, these experimental and field results suggest that *C. orientalis* can tolerate current ocean temperatures, but will have little capacity to cope with the warmer oceans projected for 2100.

The primary cause of coral bleaching is exposure to extreme ocean temperature, although longer exposure to moderate increases in temperature can also induce bleaching^46,47^. Cumulative thermal exposure is a product of the amount and the duration of stress, which is incorporated into the degree heating weeks (DHW) index, which can be used to accurately predict bleaching^3^. In the laboratory, *C. orientalis* bleached after 2.5 DHW, similar to corals that bleach after 2 DHW under natural conditions^3^. Consistent with our field observations at Orpheus Island, there are few reports of *C. orientalis* bleaching under natural conditions. In most cases, other *Cliona* species (*C. aprica*, *C. caribbaea, C. varians*, and *C. vermifera*) have tolerated periods of elevated temperature better than neighbouring corals^18–20^, including exposures exceeding 31 °C^18^ and even 33 °C^20^. In our surveys, *C. orientalis* did not bleach although temperatures did not exceed 31 °C, which is below the 32 °C thermal threshold identified during our experiment. A 32 °C threshold is consistent with other *Cliona*-*Symbiodinium* symbioses, as *C. varians* was recently reported to bleach when mean temperatures exceeded 31 °C for 10 days^48^. The combination of a 32 °C laboratory bleaching threshold with the lack of bleaching during the 2017 coral bleaching event suggests that current summer temperatures could lead to faster local erosion rates in the near future.

Coral bleaching is often preceded by disruption of *Symbiodinium* photosynthesis^6,49^ which leads to the production of toxic oxygen radicals, which must be neutralized to prevent damage to lipids, proteins and DNA^50^. The mechanisms of bleaching in sponges may be similar, however, if damage to the photosystems was responsible for triggering bleaching in *C. orientalis*, the response must have been very rapid: when *C. orientalis* bleached, *Symbiodinium* still retained ~66% of F_v_/F_m_ which had only declined for 3 days. After eight days of exposure to 32 °C, the photosynthetic capacity of the symbiosis was diminished, coinciding with a loss of *Symbiodinium* and chlorophyll. Similar effects have previously been observed in bleached *C. orientalis*^30,34^, and scleractinian corals, where a loss of *Symbiodinium* coincides with a loss of lipids, proteins, and organic matter^51,52^. In addition, bleaching can disrupt the bacterial symbioses in *C. orientalis*^53^ and scleractinian corals^54^.

Prior to *C. orientalis* bleaching, there was some evidence that respiration rates increased (29–31 °C) and that energy reserves were reduced (31 °C), suggesting that the sponges expend resources to maintain their symbiosis at sub-bleaching temperatures. Respiration in *C. orientalis* was fastest at intermediate temperatures, likely contributing to the significant decline in the productivity of the symbiosis. Nevertheless, bleached *C. orientalis* had similar respiration rates to control sponges despite their reduced condition^30^. The absence of an effect of bleaching (i.e., absence of *Symbiodinium*) on respiration rates highlights the need to separate measurement of host and *Symbiodinium* respiration^55^. Based on their low biomass relative to the biomass of the sponge tissue, it is likely that *Symbiodinium* makes a minor contribution to overall respiration, and other factors such as pumping or feeding rates may dictate energetic demand and respiration in thermally stressed sponges^56^.

The ability to persist in warming oceans will depend upon recovery of symbionts and energy reserves, before exposure to any subsequent bleaching-inducing temperatures^52^. After *C. orientalis* bleached at 32 °C, the sponges did not recover during four weeks at *30 °C, with no recovery of the symbiosis or sponge condition. The only parameter that changed during recovery was photosynthesis, where the rates of oxygen production and photochemical efficiency were higher in sponges returned to *30 °C than in sponges at 32 °C. However, based on visual observations and the lack of recovery of *Symbiodinium*, relatively high photochemical efficiency was likely due to fouling by photosynthetic epibionts rather than a re-establishment of the *Symbiodinium* population. In corals, recovery can take between 1.5 and 10 months and some species do not recover within 12 months^9,51,52,57,58^. Our experiment indicated that *C. orientalis* did not recover *Symbiodinium* under aquarium conditions, but the availability of *Symbiodinium* may have limited recovery. In other laboratory studies, *C. orientalis* have recovered *Symbiodinium* following irradiance-induced bleaching^59,60^, but further study is necessary to determine whether *C. orientalis* can regain symbionts following thermal bleaching under natural conditions. Observations in the Florida Keys, USA indicate that *C. varians* can recover from thermal bleaching (M. Hill pers. comm.), although some *Symbiodinium* likely remained within the sponge^48^.

Association with tolerant *Symbiodinium*, especially multiple types of *Symbiodinium*, can aid recovery from coral bleaching^14^. Here, all *C. orientalis* cores harboured the same symbiont, *S. endoclionum*^41^, and exhibited little flexibility in their symbiotic association before or after bleaching. This may make *C. orientalis* more vulnerable to warming than reef taxa that can associate with multiple *Symbiodinium* clades^10,12^, as *C. orientalis* harbours *S. endoclionum* over a large geographic range^41^. Little is known about the genetic diversity or physiology of clade G *Symbiodinium*, which have only have been found in bioeroding sponges^61–63^, foraminifera^64^, and one octocoral species^65^. A physiological comparison of Clade G to other *Symbiodinium* has suggested that the Clade G from *C. orientalis* are more thermally tolerant than the clade C or D inhabiting scleractinian corals^45^. However, here we have refined the thermal threshold, showing that while the clade G symbiont in *C. orientalis* can tolerate current summer temperatures (<32 °C), photosynthesis is impaired at predicted future temperatures (≥32 °C).

Recent mass coral bleaching events are a clear indication that ocean warming is a primary threat to reef corals^3^ and accelerated bioerosion by Clionaid sponges under ocean acidification would further compound the adverse outcomes of climate change. However, here we show that while the symbiosis between *C. orientalis* and its associated *Symbiodinium* tolerates current maximum sea surface temperatures, the partnership breaks down as sea surface temperatures reach 32 °C. A relatively high tolerance of present day temperature extremes may benefit *C. orientalis* via coral mortality and increased substratum availability in the short term^22,66^, however bioeroding sponges with *Symbiodinium* will be severely affected by ocean temperatures expected by 2100.

## Methods

### Laboratory experiment

Thirteen *Cliona orientalis* (Thiele, 1900) sponges were collected at 2–4 m depth from Little Pioneer Bay on Orpheus Island, Queensland, Australia (18°37′40″S, 146°29′36″E) in June 2015 (Marine Parks Permit G12/35236.1). Sponges were transported by road in 60 L plastic aquaria to the National Sea Simulator (SeaSim) at the Australian Institute of Marine Science in Townsville, Queensland, where they were maintained in outdoor flow-through aquaria at ambient temperature (23.0 °C ± 0.1 SD). After seven days in aquaria, 3.5 cm-diameter cores (n = 151) were drilled from the 13 sponges. Each sponge produced between 4 and 24 cores, depending on the size of the sponge (median = 9). Each core was labelled with the identity of the original sponge to control for genotype differences. Sixteen days after drilling, the cores from each sponge were haphazardly divided amongst nine indoor aquaria (50 L), resulting in 15–18 cores per aquarium. Ten sponges were represented in every aquarium, but three sponges were not, as they were represented by fewer than nine cores (Table 2).

Each aquarium was continuously supplied with 0.04 µm filtered seawater at 0.8 L/min. Water temperature was regulated by a SeaSim computer-controlled system to reach target temperatures; tanks were additionally buffered against temperature fluctuations using water jackets. LED lighting illuminated aquaria with 300 µmol quanta m^−2^ s^−1^ light for 11 h with an additional 1 h ramping period after dawn and before dusk. The irradiance level was less than the saturation irradiance (400 µmol quanta m^−2^ s^−1^) that was determined via rapid light curves.The irradiance level (300 µmol quanta m^−2^ s^−1^) is comparable to a cloudy day on the reef (302 µmol quanta m^−2^ s^−1^), but less than the average irradiance on a clear day (653 µmol quanta m^−2^ s^−1^) calculated from^67^. In terms of the total irradiance, irradiance in the aquaria was 12 mol d^−1^ compared to 15 or 31 mol d-1 on a cloudy or sunny day on the reef, respectively calculated from^67^. Cores were maintained at 23.2 ± 0.3 °C (±SD) for 17 days, after which sponge photosynthesis and respiration were measured and tissue samples were taken (detailed below).

To measure changes in *C. orientalis* condition and performance as a function of temperature, two temperature treatments were established: three aquaria were maintained under their initial conditions at 23 °C for the duration of the experiment (control) and six aquaria had the temperature increased every two weeks, first by 2 °C increments (23 to 29 °C) then by 1 °C increments (30 to 32 °C). Thus, the temperature targets were 23, 25, 27, 29, 30, 31, and 32 °C. These span the average range of temperatures at the collection site: 22.4 °C in July to 29.1 °C in February, with a maximum monthly mean of 30.5 °C between 2002 and 2010 (Source: Australian Institute of Marine Science; http://weather.aims.gov.au; 1.9 m depth). Each temperature increment was achieved via ramping by 0.5 °C per day up to the target temperature followed by 10 days of exposure to that temperature. The rate of temperature increase is similar to daily changes in mean temperature at the collection site calculated from^67^. The 25 °C temperature increment was extended from 14 to 24 days due to a logistical issue with the heating and cooling system. With this exception, temperatures were finely controlled throughout the experiment, typically within 0.1 °C of the target temperature and with ~0.1 °C SD among aquaria (Table 1). Exposure of sponges to these temperature increments occurred from July to November and coincided with the natural winter to summer temperature increase during the austral summer.

To evaluate the potential for *C. orientalis* to recover from bleaching, cores exposed to 32 °C were returned to 30 °C (by 0.25 °C per day) and monitored for a further 28 days. Hereafter, ‘*30 °C’ is used to distinguish the recovery period at 30 °C from the incremental increase to 30 °C. To compare the thermal exposure in the laboratory to exposure under natural conditions, degree heating weeks (DHW) of thermal exposure were calculated for each temperature target above the 29.1 + 1.0 °C bleaching threshold for Orpheus Island. DHW was calculated as the product of the °C above 30.1 °C and the duration of temperature ramping (2–4 days) and exposure to the target temperature (10 days). 30 °C was chosen as the recovery temperature as it is below the bleaching threshold and represents a typical summer temperature at the collection site.

For each temperature increment, photosynthetic measurements were taken on two days near the completion of each temperature exposure. Photochemical efficiency of all cores was measured at least twice during exposure to each increment, after one and eight days of exposure, but photochemical efficiency was measured more frequently for the 23 °C, 25 °C, 31 °C, and 32 °C temperature increments. For other photosynthetic parameters and tissue contents, 2–3 cores were selected from each tank at each temperature increment, resulting in ~6 and ~12 samples for the control and heated treatments, respectively. Oxygen flux and sponge surface area were measured after nine days at each temperature increment (due to the time required to measure oxygen flux) except for *30 °C, where measurements were taken after 28 days. After 10 days acclimation to each temperature increment (28 days at *30 °C), sponges were frozen in liquid nitrogen for DNA extraction and measurement of tissue contents.

### Photosynthesis and respiration

Photochemical efficiency is an indicator of electron transport during photosynthesis^68^. For *Symbiodinium*, decreases in photochemical efficiency can precede bleaching and coincide with damage to photosystems^69^. The photochemical efficiency of photosystem II was measured using a mini-PAM fluorometer (Walz, Effeltrich, Germany) using standard settings (MI = 8; SI = 6; SW = 0.6; G = 2; D = 2). Clear tubing was used to maintain a constant distance between the PAM fibre optic cable and the sponge. Photochemical efficiency was measured at two timepoints: before the lights turned on (F_v_/F_m_; maximum efficiency) and after two hours of constant light exposure (∆F/F_m_’; effective efficiency). Changes in these variables over the course of each temperature increment reflect the severity of the stress on the *Symbiodinium*.

Oxygen flux is an integrative measure of respiration by the sponge (and symbionts) and photosynthesis by the *Symbiodinium*^70^. For sponges, stress can manifest in altered respiration, decreased photosynthesis, or a reduced ratio of photosynthesis to respiration^29,30,71^. Oxygen flux was measured using an optical dissolved oxygen meter (Hach HQ30d; Hach, Colorado, USA). Sponge cores were sealed in a darkened chamber (500 mL) for one hour. Water within each chamber was mixed with a magnetic stir bar and the target temperature was maintained using an external water jacket. Respiration rates were calculated by the difference in oxygen concentration between the beginning and end of the incubations. After measurement of respiration, sponges were returned to the aquaria for one hour at 300 µmol quanta m^−2^ s^−1^ then sealed in a chamber at 400 µmol quanta m^−2^ s^−1^ to measure oxygen production. The photosynthetic rate was calculated using the change in oxygen concentration in the chamber over 40 minutes. Both respiration and photosynthetic rates were adjusted by the amount of oxygen flux in a chamber without a sponge core to account for oxygen flux by microorganisms in the water. The surface area of the selected cores was measured using the aluminium foil method^72^, sponge tissue was removed (top 1 cm), the cores were frozen in liquid nitrogen and stored at −80 °C.

### Sponge condition (*Symbiodinium*, chlorophyll, protein, organic content)

Invertebrates that harbour *Symbiodinium* can bleach by losing symbionts or their photosynthetic pigments^73^. *Symbiodinium* cells were extracted by incubating sponge tissue in 1 M NaOH at 37 °C for 1 h^74^. Cells were counted using 4 replicate counts of 0.45 cm^2^ on a hemocytometer and standardised to the wet weight of sponge tissue. To quantify the photosynthetic pigments within *C. orientalis* tissues, chlorophylls were extracted in two consecutive extractions of 1 mL of ethanol (95%) to ensure complete extraction of pigments. During each extraction, the tissue was homogenized for 3 min in a bead beater, centrifuged for 5 min (10,000 g), after which the two extracts were pooled. Absorbance of the extract was measured at 630, 647, 664, and 750 nm using a Power Wave Microplate Scanning Spectrophotometer (BIO-TEK Instruments Inc., Vermont, USA). The concentrations of chlorophylls *a*, *b*, and *c* were estimated using the equations of Ritchie^75^ and standardised to sponge wet weight.

Protein content and tissue organic matter were measured as proxies for sponge condition as these parameters have been shown to decline in bleached corals^51^. Frozen sponges were weighed (wet weight), lyophilized, and weighed again (dry weight). Proteins were extracted from homogenized dried sponge in 2 mL of 0.125 M NaOH over 24 h at room temperature. Protein concentration was estimated using the Red660 Protein Assay with five concentrations of BSA protein standard and then standardised to the dry weight of the sponge tissue. Organic matter was measured using lyopholized sponge tissue (see Protein content). Dried sponge was combusted at 450 °C for 16 h. Organic matter was estimated as the difference between the dry weight and the ash weight and standardised to the dry weight of the sponge.

### *Symbiodinium* identity

To determine whether the sponges switched *Symbiodinium* types during temperature stress, we identified the *Symbiodinium* associated with cores from 9 of the original sponge fragments as temperatures increased. In total, 35 cores were analysed including cores from the same genotype in each temperature increment. DNA was extracted from frozen sponge tissue (~0.2 g) using the Powerplant Pro DNA Isolation Kit (Mo Bio), including the beadbeating, RNAse, and proteinase K procedures as per the manufacturer’s instructions. DNA extracts were sent to the Australian Centre for Ecogenomics at the University of Queensland, Australia for sequencing. The ITS2 region of ribosomal rDNA was amplified using *Symbiodinium*-specific ITS2 primers^76^ and sequenced using Illumina MiSeq250 bp chemistry.

Sequences were analysed in Mothur v.1.38.0^77^. Paired reads were combined and screened for quality and chimeric sequences were identified using Uchime in Mothur. The remaining sequences were clustered into 97% similar operational taxonomic units (OTU). The dataset was reduced to 2000 sequences per sample and the relative abundance and prevalence were calculated for each OTU. Representative sequences were defined using the sequence with the smallest distance to all other sequences within the OTU. Sequences were compared against a curated database of ITS2 sequences including all clades of *Symbiodinium* using the BLAST algorithm^78^. Blast results with bit scores less than 100 were discarded.

### Bleaching surveys

Field surveys were conducted to assess the thermal tolerance of *C. orientalis* during a natural thermal bleaching event, and to contrast bleaching responses between *C. orientalis* and corals. In March 2017, video transects were filmed at six sites within the Palm Islands Group, three of which were at Orpheus Island where the experimental samples were collected. Survey sites were chosen as replicate exposed and protected locations, as well as to span the depth gradient over which *Acropora* cover is relatively high at Orpheus Island. At each site, two transects (50 m long and parallel to shore) were filmed at 0–4 m below the lowest astronomical tide. *Cliona orientalis* sponges and scleractinian coral colonies were manually counted along each video transect. Coral colonies were categorized as either branching or massive and we used a simple ‘bleached’ or ‘unbleached’ categorisation due to the absence of a reliable colour reference in the videos. White individuals, as well as individuals with fluorescent discolouration (e.g., blue or pink Acropora spp.) were considered bleached.

To compare the thermal exposure during the natural bleaching event to the laboratory experiment, daily mean and maximum temperatures at Orpheus Island (5.8 m depth) were downloaded from the Australian Institute of Marine Science (http://weather.aims.gov.au).

### Statistical analysis

Photosynthesis and sponge condition data were analysed using linear mixed models with treatment, time point, and treatment * time point interaction as well as a random intercept to account for between-sponge differences using the R packages lme4^79^, lmerTest^80^, and multcomp^81^. Only the final photochemical efficiency was analysed statistically. *Symbiodinium* density was analysed with an additional random intercept to account for correlations between samples from the same aquarium. Boxplots and residual plots were used to assess whether the data met the assumptions of linear models. Samples with large deviations from fitted values (>1.5*interquartile range) were removed from the analysis. Some response variables were transformed using log (respiration, chl *a*:*c*, protein, organic matter) or odds ratios (F_v_/F_m_, ∆F/F_m_’) to meet these assumptions. Planned contrasts were used to test whether heated sponges differed from control sponges at each time point, as well as whether changes occurred after the reduction in temperature following exposure to 32 °C. Results from the planned contrasts are reported with z statistics and *P* values. *P* values were corrected using a single-step correction for multiple comparisons.

For each benthic category in the bleaching surveys, individuals were pooled between replicate transects at each site. The proportion of bleached individuals was calculated out of the total number of individuals encountered at each site. For each category of taxa, the proportion of bleached individuals was calculated using proportion of bleached individuals relative to the total number of colonies and then weighted according to the number of individuals at each site.

### Data availability

Data are available from the authors upon request.
